# The Jubilee of the American Institute

**Published:** 1894-03

**Authors:** Pemberton Dudley

**Affiliations:** General Secretary; 1405 North Sixteenth Street, Philadelphia, Pa.


### THE JUBILEE OF THE AMERICAN INSTITUTE.

The Executive Committee of the American Institute of Homœopathy has named Thursday, June 14th, 1894, as the time for the opening of the next annual session. Physicians starting from the most distant points on Sunday evening can reach Denver by Thursday morning. The order of business is not yet arranged, but it has been suggested that the session open at three o'clock P. M., that the afternoon be devoted to general routine business, and that the Special Jubilee Exercises and the delivery of the President's Address take place in the evening.

Under the new By-Laws the duration of the session will be limited only by the needs of the business and the requirements of the Sections; each of the latter being allowed all the time its members may desire for the reading and discussion of all its papers. Essayists are thus assured that their papers will in no instance be denied a respectful hearing for want of time, and the specialists of the Institute can enjoy full opportunity for the consideration of the technical questions in which they may be interested. Illustrations intended for publication in the Transactions should be artistically made and on separate sheets for the use of the engraver. The Institute does not object to a reasonable expense when necessary in illustrating an essay. The value and interest of the scientific discussions will be greatly enhanced if each essayist will furnish copies of his paper prior to the session to those who are expected to lead in debating it.

Any physician having knowledge of the decease of an Institute member since June 1st, 1893, will confer a favor by reporting full particulars to the Necrologist, Dr. Henry M. Smith,

Spuyten Duyvil, New York. Secretaries or other officers of all societies, clubs, hospitals, dispensaries, etc., and the physicians of all institutions of whatsoever kind employing homœopathic treatment are earnestly requested to make full reports to Dr. T. Franklin Smith, Chairman of the Committee on Organizations, etc., 264 Lenox Avenue, New York City, who will furnish blanks for that purpose.

The Annual Circular, with full particulars as to hotels, railroad fares, programme, and other matters of interest, will be mailed in May to every homœopathic physician in the United States and Canada. Any physician failing to receive it by May 20th should notify the Secretary. Each circular will contain a blank application for membership, with full directions for those desiring to become members. Societies and colleges wishing to canvass their membership for new members of the Institute should apply at once for blanks, stating the number desired.

During the last six years the Institute membership has grown from 900 to 1,613—about 80 per cent. It was suggested at the last session that each member should celebrate the Jubilee by securing at least one new member for the meeting at Denver.

PEMBERTON DUDLEY, M. D.,
*General Secretary.*

1405 North Sixteenth Street, Philadelphia, Pa.

